# Acute radiation-induced pericarditis complicated by polymicrobial infectious pericarditis in a patient with mediastinal sarcoma: a case report

**DOI:** 10.1093/ehjcr/ytae084

**Published:** 2024-02-09

**Authors:** Kimberly L Yan, Yoo Jin Lee, Alan H Baik

**Affiliations:** Department of Medicine, University of California, San Francisco, 505 Parnassus Ave, San Francisco, CA 94143, USA; Department of Radiology, University of California, San Francisco, San Francisco, CAUSA; Department of Medicine, University of California, San Francisco, 505 Parnassus Ave, San Francisco, CA 94143, USA; Division of Cardiology, Section of Cardio-Oncology and Immunology, University of California, San Francisco, 555 Mission Bay Blvd South, San Francisco, CA 94158, USA

**Keywords:** Acute pericarditis, Cardiac tamponade, Radiation toxicity, Cardio-oncology, Polymicrobial infectious pericarditis, Case report

## Abstract

**Background:**

Acute pericarditis is often caused by viral infections, autoimmune diseases, and radiation therapy (RT). Infectious pericarditis is rare and associated with high morbidity and mortality. We present a case of acute RT-induced pericarditis complicated by bacterial pericarditis and cardiac tamponade due to oesophageal bacterial translocation.

**Case summary:**

A 65-year-old man with a recurrent mediastinal sarcoma complicated by oesophageal compression and recent oesophageal stenting presented with shortness of breath. Electrocardiogram showed diffuse ST elevations, and he was diagnosed with presumed RT-induced pericarditis. Despite anti-inflammatory therapy, he developed haemodynamic instability and clinical tamponade, with transthoracic echocardiogram showing a large circumferential pericardial effusion. He underwent emergent pericardiocentesis, and pericardial fluid cultures grew polymicrobial species. Anti-inflammatories were held, and he was started on broad spectrum intravenous antibiotics and antifungals. Due to clinical decompensation and repeat computed tomography imaging demonstrating worsening pericardial disease, he underwent pericardial irrigation and subxiphoid pericardial window. The patient died from hypoxaemic and hypercapnic respiratory failure. Autopsy revealed constrictive pericarditis and no bacterial organisms in the pericardium.

**Discussion:**

Anti-inflammatories are standard treatment for viral and RT-induced pericarditis. Purulent, bacterial pericarditis is rare and an uncommon complication of RT-induced pericarditis. Polymicrobial infectious pericarditis is often refractory to intravenous antibiotics, requiring surgical intervention. This case highlights the importance of maintaining a high index of suspicion of various potential aetiologies of pericarditis in order to tailor medical and surgical therapies especially in high-risk, immunosuppressed cancer patients.

**Learning points**

- Pericardiocentesis and pericardial fluid analysis are important in the diagnosis of pericarditis that does not improve with standard anti-inflammatory therapy.
- Bacterial pericarditis is a rare cause of acute pericarditis secondary to intrathoracic infections, haematogenous spread, or direct infection. It should be considered in patients with high-risk mediastinal sarcoma following radiation therapy.

## Introduction

Acute pericarditis is often caused by viral infections, autoimmune disease, and radiation therapy (RT) and is typically treated with anti-inflammatory agents.^1^ Bacterial pericarditis is a rare cause of acute pericarditis secondary to intrathoracic infections, haematogenous spread, or direct infection via trauma or surgery.^2,3^ Patients with thoracic malignancies often receive mediastinal RT which can cause adverse cardiovascular effects. Acute RT-induced pericarditis is common, but it has rarely been reported to co-occur with polymicrobial bacterial pericarditis and tamponade. The aim of this case is to describe acute pericardial complications of radiation therapy, the management of these adverse cardiac events, and the pathology findings from radiation pericarditis complicated by bacterial pericarditis.

## Summary figure

**Table:** 

Date	Event
**22–24 June 2022**	Sessions 1–3 of 10, planned total 40 Gy external beam radiation for mediastinal sarcoma
**26 June 2022**	Admitted for acute pericarditis secondary to chest radiation Electrocardiogram: sinus tachycardia, diffuse ST segment elevations, and knuckle sign in lead aVR, consistent with acute pericarditis Transthoracic echocardiography (TTE): new moderate to large circumferential pericardial effusion with fibrinous strands present and no tamponade physiology Initiated on standard anti-inflammatories [high-dose non-steroidal anti-inflammatory drugs (NSAIDs) and colchicine]
**27 June 2022**	Developed haemodynamically significant atrial fibrillation with rapid ventricular response Non-steroidal anti-inflammatory drugs discontinued. Prednisone started for suspected refractory acute radiation therapy (RT)-induced pericarditis
**28 June 2022**	Developed clinical tamponade Transthoracic echocardiography: large circumferential pericardial effusion with echocardiographic signs of tamponade Subcostal pericardiocentesis with pericardial drain placement
	Computed tomography (CT) showing simple pericardial effusion (arrows) with pericardial drain in place, 6 days after mediastinal RT
**30 June 2022**	Pericardial fluid from 6/28 growing *Klebsiella pneumoniae*, *Escherichia coli*, *Pseudomonas aeruginosa* Stopped prednisone; initiated IV vancomycin/ceftriaxone
**1 July 2022**	Vancomycin/ceftriaxone narrowed to piperacillin/tazobactam/fluconazole based on culture susceptibilities including *Candida glabrata*
**4 July 2022**	Fluconazole changed to micafungin due to QT prolongation
**11 July 2022**	CT chest showing increased, complex gaseous and heterogeneous pericardial effusion, pericardial thickening (arrows), and pneumopericardium, 21 days after mediastinal RT
**12 July 2022**	Pericardial irrigation and subxiphoid pericardial window with pericardial drain placement; operative pericardial fluid bacterial culture growing multi-organism species
**20 July 2022**	Death

## Case presentation

**Figure 1:**
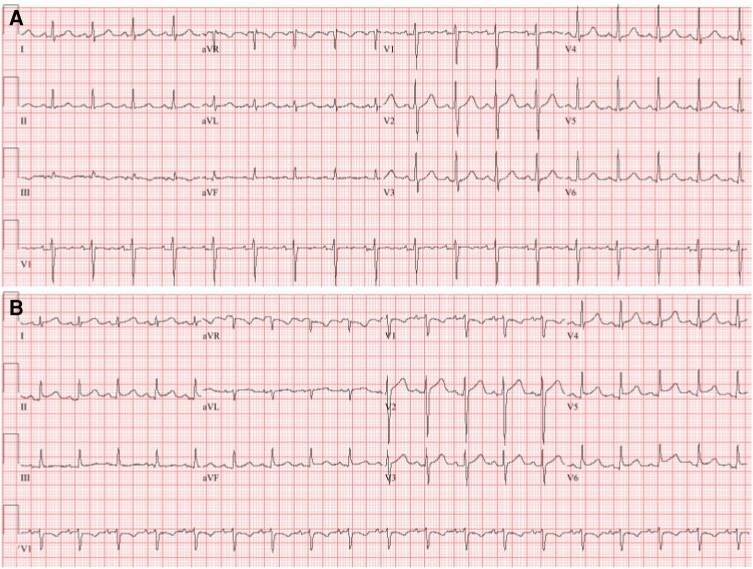
Electrocardiogram consistent with acute pericarditis. (*A*) Baseline electrocardiogram prior to radiation therapy showing sinus tachycardia. (*B*) Electrocardiogram following the third consecutive day of mediastinal radiation therapy showing sinus tachycardia with diffuse ST segment elevations and knuckle sign in lead aVR, consistent with acute pericarditis.

**Figure 2:**
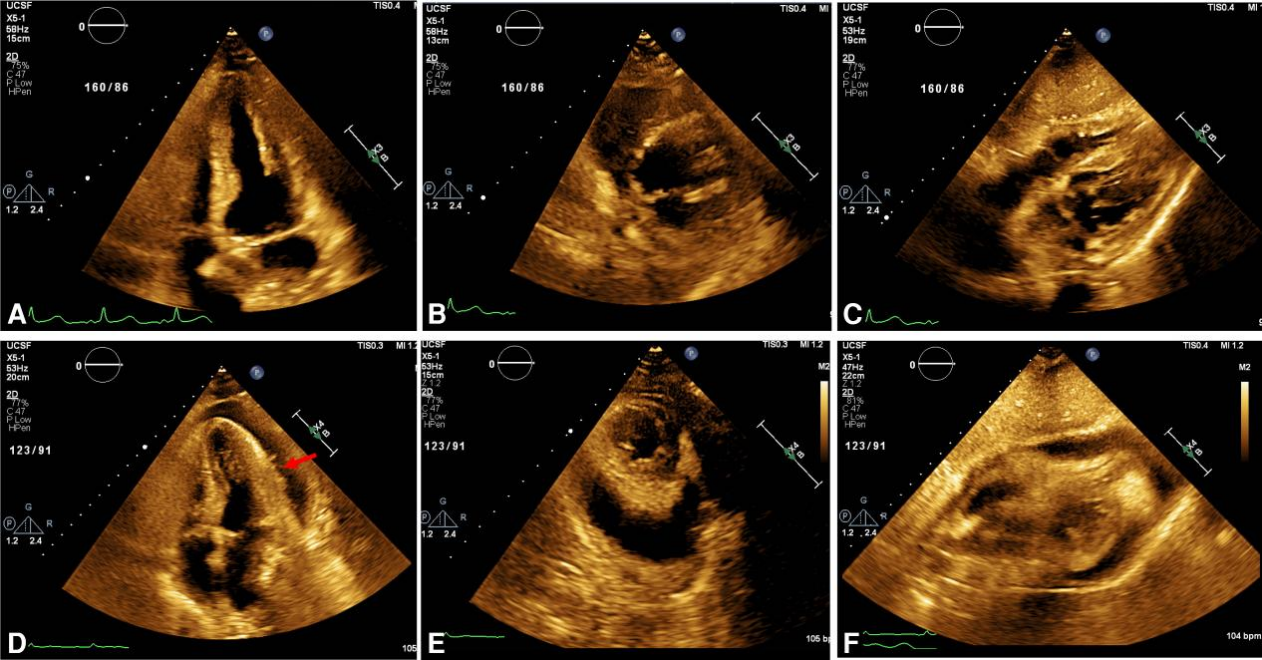
Transthoracic echocardiogram with new moderate pericardial effusion. Baseline pre-radiation therapy (RT) transthoracic echocardiogram without evidence of pericardial effusion: (*A*) apical four-chamber view, (*B*) short-axis view, and (*C*) subcostal view. Transthoracic echocardiogram 6 days after RT showing a new circumferential moderate pericardial effusion with fibrinous strands (arrow). There was no echocardiographic evidence of tamponade: (*D*) apical four-chamber view, (*E*) short-axis view, and (*F*) subcostal view.

We present a 65-year-old man with a recurrent posterior mediastinal sarcoma complicated by oesophageal compression and treated with surgical resection, oesophageal stenting, neoadjuvant chemotherapy [doxorubicin (total cumulative dose of 150 mg/m^2^) and ifosfamide], and RT (cumulative dose of 50 Gy). Due to recurrence of the sarcoma (27 × 52 mm), he underwent a second round of RT with curative intent. Baseline electrocardiogram (ECG) showed sinus tachycardia (*Figure 1A*). There was no pericardial effusion on transthoracic echocardiography (TTE) obtained 14 days prior to RT (*Figure 2A–C*). One day following his third subsequent dose of RT, he developed acute onset dyspnoea. The patient was afebrile, with a heart rate of 120 b.p.m. and blood pressure 130/96 mmHg. Exam was notable for a pericardial friction rub. Jugular venous distension was absent. Labs were notable for a white blood cell count of 11 900 white blood cells (WBCs)/µL, erythrocyte sedimentation rate of 42 mm/h, and C-reactive protein of 262 mg/L. Electrocardiogram showed sinus rhythm with diffuse ST segment elevations and knuckle sign (PR elevation and ST depression) in aVR (*Figure 1B*). Transthoracic echocardiography demonstrated a new circumferential moderate pericardial effusion with fibrinous strands. There was no echocardiographic evidence of tamponade (*Figure 2D–F*). He was diagnosed with acute RT-induced pericarditis and treated with colchicine and high-dose ibuprofen.

**Figure 3:**
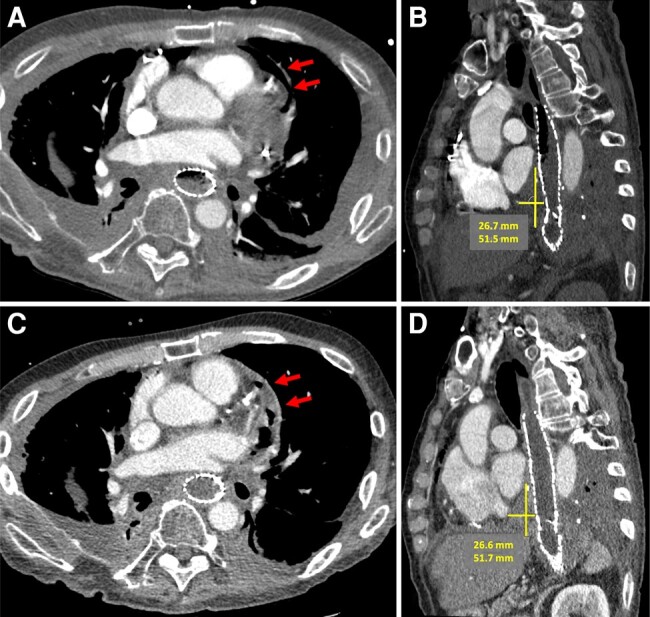
Serial CT imaging of mediastinal sarcoma and pericardium. (*A*) Axial view, CT angiogram of chest showing simple, homogenous pericardial effusion (arrows) and pneumopericardium with pericardial drain in place, 6 days after mediastinal RT. (*B*) Sagittal view, CT chest demonstrating a 27 × 52 mm posterior mediastinal sarcoma (margins demarcated by lines) exerting mass effect to the left atrium and oesophagus (status post oesophageal stent), 6 days after mediastinal RT. (*C*) Axial view, repeat CT with contrast showing increased, complex gaseous and heterogeneous pericardial effusion, pericardial thickening (arrows), and pneumopericardium, 21 days after mediastinal RT. (*D*) Sagittal view, repeat CT chest redemonstrating similar sized sarcoma and thickened pericardium, 21 days after mediastinal RT.

Two days later, he developed atrial fibrillation with rapid ventricular response associated with haemodynamic instability, which responded to intravenous amiodarone and fluids. He was started on oral prednisone for refractory RT-induced pericarditis. Exam was notable for a pulsus paradoxus of 16 mmHg and elevated JVP, consistent with acute tamponade. The patient underwent emergent pericardiocentesis revealing a serosanguineous, exudative effusion. Cytology of the pericardial fluid was negative for malignancy. However, cultures grew polymicrobial species (*Klebsiella pneumoniae*, *Escherichia coli*, *Pseudomonas aeruginosa*), suspicious for a gastrointestinal source. Intravenous vancomycin and ceftriaxone were initiated and then narrowed to piperacillin/tazobactam/fluconazole based on culture susceptibilities including *Candida glabrata*. Anti-inflammatories were discontinued. Chest computed tomography (CT) imaging demonstrated a normal-appearing pericardium, a homogenous pericardial effusion, and pneumopericardium with pericardial drain, 6 days after first mediastinal RT (*Figure 3A* and *B*). The pericardial drain was removed 6 days after initial placement when the drain output was <50 cc over 24 h.

Despite 2 weeks of IV antibiotics, repeat CT imaging 21 days after RT demonstrated pneumomediastinum and worsening loculated pericardial effusion and pericardial thickening, raising concern for inadequate source control of the infection or oesophageal–pericardial fistula (*Figure 3C* and *D*). Serial CT and fluoroscopic oesophagrams were negative for oesophageal–pericardial fistula. Bacteraemia and fungaemia were ruled out with serial negative central and peripheral blood cultures. He underwent pericardial irrigation and subxiphoid pericardial window, revealing a thick fibrinous exudate. The pericardial cavity was irrigated with saline and antibiotics, and another pericardial drain was placed. No fibrinolytics were used. Pathology from the pericardium showed fibrinous debris with mixed acute and chronic inflammation and granulation tissue. Bacterial and fungal cultures from the pericardial fluid showed rare multi-organism species. The patient died on hospital day 26 due to hypoxaemic and hypercapnic respiratory failure secondary to RT-induced pneumonitis and pulmonary fibrosis.

**Figure 4:**
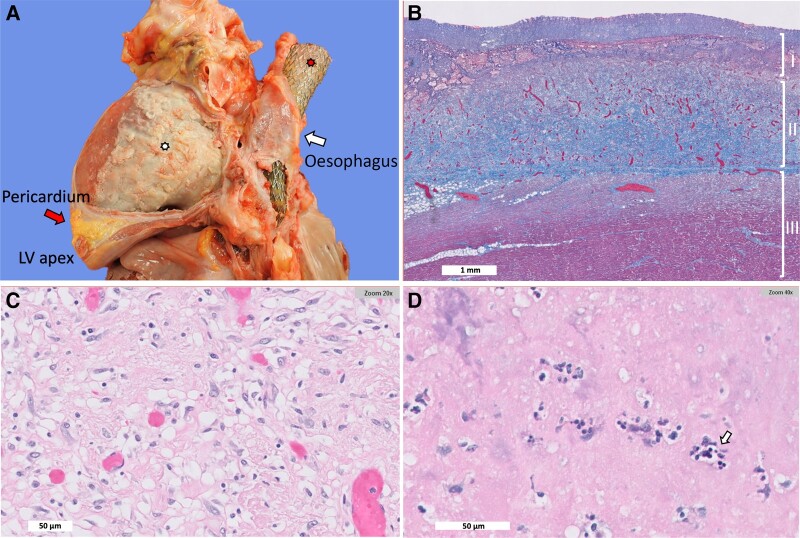
Autopsy and pathology findings from myocardium and pericardium. (*A*) Gross specimen of the heart and oesophagus demonstrating dense fibrous material over the surface of the anterior aspect of the left ventricle (bottom asterisk), thickened pericardium (left arrow), large oesophageal stent (top asterisk), and oesophagus (right arrow). (*B*) Gomori trichrome stain of pericardium and myocardium demonstrating myocardium (Layer III), organizing pericarditis with granulation tissue (Layer II), and superimposed acute pericarditis with fibrinous exudate (Layer I). (*C*) Middle layer of pericardial granulation tissue with fibroblasts and capillaries as well as scattered macrophages; haematoxylin and eosin (H&E) stain. (*D*) Top layer of the pericardium showing fibrinous exudate with occasional neutrophils (arrow) on H&E stain. Gram stain did not show evidence of bacterial organisms (not shown).

Autopsy revealed a CD34-positive posterior mediastinal sarcoma involving the anterior wall of the oesophagus with extension in the posterior pericardium. There was no evidence of an oesophageal–pericardial fistula. Autopsy was consistent with a fibrinous organizing pericarditis with adhesions to the diaphragm and left lung, in addition to RT-induced organizing pleuritis and radiation pneumonitis (*Figure 4A* and *B*). Microscopic findings demonstrated subendocardial myocytolysis of both ventricles, presence of intra-alveolar macrophages, and interstitial fibrosis of the lung parenchyma. Trichrome staining of the heart, Elistachrome of the lungs, and CD34 staining of both demonstrated RT-associated changes and no evidence of sarcoma in the heart, lungs, or pleura. Haematoxylin and eosin (H&E) staining of the middle and outermost layers of pericardium showed granulation tissue rich with fibroblasts and fibrinous exudate with occasional neutrophils, respectively (*Figure 4C* and *D*). No bacterial microorganisms were detected on Gram bacterial stain performed on the pericardium. Ultimately, the cause of death was attributed to severe complications from radiation including pericarditis, pleuritis, and pneumonitis.

## Discussion

Here, we present a rare case of RT-induced acute pericarditis complicated by polymicrobial bacterial pericarditis and tamponade in a patient with a recurrent mediastinal sarcoma. Cancer patients who receive mediastinal RT can develop adverse cardiac events. Acute RT toxicities include pericarditis, pericardial effusions, and arrhythmias. Acute pericarditis is the most common pericardial cardiotoxicity, and diagnosis requires two of the four characteristics: pleuritic chest pain, pericardial friction rub, ECG with diffuse ST elevations and PR depressions, and new or worsening pericardial effusion on echocardiogram. Additional supporting diagnostic criteria include elevated inflammatory markers and a positive pulsus paradoxus in the presence of tamponade.^4^ In this case, the patient was initially diagnosed with acute pericarditis secondary to mediastinal RT based on his classic ECG changes, pericardial friction rub on exam, and new pericardial effusion on TTE.

The pathophysiology of RT-induced acute cardiac disease involves the activation of acute inflammatory pathways, leading to cytokine release and oxidative stress.^5^ Acute inflammation can trigger cellular dysfunction and ischaemia resulting in fibrin deposition and fibrosis, which can lead to chronic toxicities, including constrictive pericarditis, valvular disease, arrhythmias, and coronary artery disease.^5^ In this patient, autopsy that was performed 4 weeks after mediastinal radiation showed a fibrinous organizing constrictive pericarditis with adhesions to the diaphragm, supporting a subacute process, consistent with radiation pericarditis with fibrin deposition and pericardial thickening. The pericardium did not have evidence of scar or dense fibrosis, which can develop from chronic pericardial inflammation and lead to constriction. There were no bacterial organisms or neutrophils in the pericardium’s outermost fibrinous layer. The chest imaging from this case also demonstrates progression of RT pericardial changes that can result when anti-inflammatories are held due to bacterial super-infection.

Anti-inflammatories, including high-dose non-steroidal anti-inflammatory drugs (NSAIDs) and colchicine, are the first-line treatment for acute pericarditis (Class I recommendation from the 2015 European Society of Cardiology guidelines). Corticosteroids can be considered in refractory cases. However, anti-inflammatories have no defined role in bacterial pericarditis. Purulent, bacterial pericarditis is rare and associated with high morbidity and mortality. It can occur in immunocompromised patients and typically results from haematogenous spread, trauma, or following thoracic surgery. Bacterial pericarditis is associated with oesophageal cancer and oesophageal–pericardial fistula, which was not observed in this patient’s autopsy findings.^6,7^ This case demonstrates that bacterial pericarditis can result from bacterial translocation from the oesophagus secondary to RT-induced inflammation. Pericardial drainage and cultures are critical for those with high-risk factors (i.e. immunocompromised and presence of tamponade) because of the urgency of antibiotic initiation and management.^8^ Purulent pericarditis can only be definitely diagnosed with pericardial fluid bacterial cultures and fluid analysis, which is typically exudative with low glucose, high protein and lactate dehydrogenase fluid to serum ratios, and elevated neutrophils. Involvement of infectious disease specialists is also recommended in the management of purulent pericarditis.

Bacterial pericarditis is often refractory to antibiotics and can lead to acute tamponade and constrictive pericarditis, which can affect 20–30% of patients with purulent pericarditis.^9^ Indeed, in this case, the patient developed a rapidly growing pericardial effusion with clinical tamponade, requiring emergent pericardiocentesis due to polymicrobial bacterial pericarditis. Treatment requires pericardial irrigation and pericardiectomy, especially in cases of refractory constrictive pericarditis. Source control is of paramount importance in purulent, infectious pericarditis, which can be difficult to achieve in high-risk, immunosuppressed cancer patients.

## Data Availability

All relevant patient data and imaging are presented in the main manuscript and figures.

## Lead author biography

Kimberly L. Yan is a second-year internal medicine resident at University of California, San Francisco (UCSF). She completed her medical training at the David Geffen School of Medicine at University of California, Los Angeles. She plans to continue her training in cardiology fellowship.
